# Supplementary material for: Immunological Fluid Biomarkers in Frontotemporal Dementia: A Systematic Review
Source: Biomolecules. 2025 Mar 24;15(4):473. doi: 10.3390/biom15040473 (PMC12025258; doi:10.3390/biom15040473)
Supplement: Supplementary file 1 [file biomolecules-15-00473-s001.zip › Supplementary table S1 - QUADAS-2.pdf]

Adjusted QUADAS-2 for Risk of Bias Assessment

This systematic review examines immunological biomarkers in patients with frontotemporal dementia. As the primary focus of the study is on biomarker quantification and comparison rather than diagnostic accuracy, the QUADAS-2 tool was adapted accordingly. The modified QUADAS-2 framework, outlined below, was utilized to assess the risk of bias and concerns regarding applicability.

QUADAS-2 Domains and Signaling Questions

All signaling questions are answered with 'Yes', 'No', or 'Unclear' based on the available information in each study.

1. Patient Selection

- **Risk of Bias Signaling Questions:**
    1. Were FTD patients diagnosed based on internationally recognized diagnostic criteria (e.g., Raskovsky et al. 2011, Gorno-Tempini et al. 2011)?
    2. If not, were FTD patients selected using clearly defined inclusion/exclusion criteria?
    3. Was the clinical or genetic subtype of FTD patients reported?
- **Applicability Concerns:**
    - Were the included FTD patients representative of the broader FTD population?
    - Were the control groups appropriate for the study objective?

2. Index Test (Biomarker Measurement)

- **Risk of Bias Signaling Questions:**
    1. Was the immunological biomarker measured using a validated and standardized assay (e.g., ELISA, Simoa)
    2. Was the same assay method consistently applied to both FTD patients and control groups?
    3. Were there replicates or quality control measures used in the biomarker measurements?
    4. Was all data, including non-significant results, reported transparently?
    5. Were statistical methods clearly described and suitable for the study design?
- **Applicability Concerns:**
    - Were the chosen biomarkers supported by previous research showing their relevance to immune dysfunction in FTD, including at least three independent publications demonstrating their direct effect on relevant immunological components (e.g., inflammation, immune cells, cytokines)?"
    - Was the biomarker measurement method appropriate for FTD-related immune analysis?
    - Were the reported biomarkers relevant to immune dysregulation in FTD?

3. Reference Standard (Control Group Definition and Comparison)

- **Risk of Bias Signaling Questions:**
    1. Was the control group (healthy individuals or neurological controls) appropriately selected and well-defined?
    2. Were control subjects assessed using appropriate diagnostic criteria to confirm their classification?
    3. Were demographic characteristics (e.g., age, sex) comparable between FTD and control groups?
    4. Were control groups balanced with respect to important confounders (e.g., comorbidities, medications)?
- **Applicability Concerns:**
    - Were the selected control groups suitable for biomarker comparisons with FTD patients?
    - Were the control groups reflective of the general population or the broader group of neurological disorders?

4. Flow and Timing (Sample Handling and Data Integrity)

- **Risk of Bias Signaling Questions:**
    1. Were pre-analytical conditions (e.g., storage, freeze-thaw cycles, handling) clearly reported and consistent across groups?
    2. Were all relevant data (e.g., sample size, missing data) accounted for in the final analysis?

Risk of Bias Assessment Guide

- **Low Risk of Bias**: If the study met all criteria relevant to the domain.
- **High Risk of Bias**: If one or more criteria indicated methodological weaknesses.
- **Unclear Risk of Bias**: If the study did not report enough details to assess the domain.

Risk of Bias and Applicability Concerns Assessment for Included Studies

Study (PMID)	Risk of bias				Applicability concerns		
	Patient selection	Index test	Reference standard	Flow and timing	Patient selection	Index test	Reference standard
Abu-Rumeileh et al. (31892365)	Low	Low	Low	Low	Low	Low	Low
Abu-Rumeileh et al. (31957347)	Low	Low	Low	Low	Low	Low	Unclear
Alcolea et al. (24820015)	Low	Low	Low	Low	Low	Low	Low
Alcolea et al. (28592456)	Unclear	Low	Low	Low	Low	Low	Low
Alexopoulos et al. (22045485)	Low	Low	Low	Low	Low	Low	Low
Andrés-Benito et al. (33213069)	Low	Low	Low	Low	Low	Low	Low
Antonell et al. (31668967)	Low	Low	Low	Low	Low	Low	Low
Arshad et al. (34237731)	Low	Low	Low	Unclear	Low	Low	Low
Asken et al. (36977552)	Low	Low	Low	Low	Low	Low	Low
Baiardi et al. (36221099)	Low	Low	Low	Low	Low	Low	Low
Beers et al. (32943739)	High	High	High	High	High	Low	High
Bellini et al. (35159297)	Low	Low	Low	Low	Low	Low	Low
Bergström et al. (34838088)	Low	Low	Low	Low	Low	Unclear	Low
Blasko et al. (16244482)	Low	Low	Low	Low	Low	Low	Low
Blauwendraat et al. (28749476)	Low	Low	Low	Low	Unclear	Low	Low
Borrego-Écija et al. (38018380)	Low	Low	Low	Low	Low	Low	Unclear
Bossolasco et al. (28427413)	Low	Low	Low	Low	Low	Low	Low
Bossù et al. (21645364)	Low	Low	Low	Unclear	Low	Low	Low
Braun et al. (37833765)	Low	Low	Low	Low	Low	Low	Low
Bruggink et al. (26119087)	Low	Low	Low	Low	Low	Low	Unclear
Busse et al. (28582858)	High	Low	Low	Unclear	High	Low	Low

Study (PMID)	Risk of bias				Applicability concerns		
	Patient selection	Index test	Reference standard	Flow and timing	Patient selection	Index test	Reference standard
Busse et al. (34589754)	Unclear	Low	Low	Unclear	Low	Low	Low
Böstrom et al. (33814444)	Low	Low	Low	Low	Low	Low	Low
Cavazzana et al. (30391902)	Low	Low	Low	Unclear	Low	Low	Low
Chen et al. (38588788)	Low	Low	Low	Low	Low	Low	Low
Chouliaras et al. (35078917)	Low	Low	Low	Low	Low	Low	Low
Chu et al (36890594)	Low	Low	Low	Low	Low	Low	Low
Çoban et al. (23813609)	Low	Low	Low	Unclear	Low	Low	Unclear
Comi et al. (20308780)	Low	Low	Low	Low	Low	Low	Low
Conti et al. (33479441)	Low	Low	Low	Low	Low	Low	Unclear
Craig-Schapiro et al. (21035623)	Low	Low	Low	Low	Low	Low	Low
de Luna et al. (36460480)	Low	Unclear	Low	Unclear	Low	Low	Low
Del Campo et al. (30349851)	Low	Low	Low	Low	Low	Low	Low
Derkow et al. (30011310)	Low	Low	Low	Low	Low	Low	Unclear
Dols-Icardo et al. (22502998)	Low	Low	Low	Unclear	Low	Low	Low
Ernst et al. (17698209)	Low	Low	Low	Low	Low	Low	Low
Feneberg et al. (26659729)	Low	Low	Low	Low	Low	Low	Unclear
Fenoglio et al. (39153518)	Low	Low	Low	Low	Low	Low	Low
Finch et al. (19158106)	Low	Low	Low	Unclear	Low	Low	Unclear
Fraga et al. (34656268)	Low	Low	Low	Unclear	Low	Low	Low
Galimberti et al. (16401871)	Low	Low	Low	Unclear	Low	Low	Low
Galimberti et al. (18204920)	Low	Low	Low	Low	Low	Low	Low
Galimberti et al. (19494437)	Low	Low	Unclear	Low	Low	Low	Low
Galimberti et al. (26021560)	Low	Low	Low	Low	Low	Low	Low
Ghidoni et al. (18768919)	Low	Low	Low	Unclear	Low	Low	Unclear

Study (PMID)	Risk of bias				Applicability concerns		
	Patient selection	Index test	Reference standard	Flow and timing	Patient selection	Index test	Reference standard
Ghidoni et al. (24018267)	Low	Low	Low	Unclear	Low	Low	Low
Gibbons et al. (25435337)	Low	Low	Unclear	Low	Low	Low	Low
Gomez-Tortosa et al. (23724906)	Low	Low	Low	Low	Low	Low	Unclear
González-Sánchez et al. (32276479)	Low	Low	Low	Low	Low	Low	Low
Heikkinen et al. (37038815)	Low	Low	Low	Unclear	Low	Low	Low
Heywood et al. (30292090)	Low	Low	Low	Low	Low	Low	Unclear
Hu et al. (21048198)	Low	Low	Low	Low	Low	Low	Low
Hu et al. (24174584)	Low	Low	Low	Low	Low	Low	Low
Illán-Gala et al. (30291183)	Low	Low	Low	Unclear	Low	Low	Low
Ishiki et al. (26485083)	Low	Low	Low	Unclear	Low	Low	Unclear
Janelidze et al. (26783546)	Low	Low	Low	Low	Low	Low	Low
Janelidze et al. (28061383)	Low	Low	Low	Low	Low	Low	Low
Janssen et al. (14991353)	Low	Low	Low	Unclear	Low	Unclear	Low
Jeppson et al. (31167811)	Low	Low	Low	Low	Low	Low	Low
Jesse et al. (21188408)	Low	Unclear	Low	Low	Low	Low	Unclear
Jääskeläinen et al. (31561355)	Low	Low	Low	Low	Low	Low	Low
Kankapaa et al. (19130885)	Low	Low	Low	Low	Low	Low	Low
Katisko et al. (30320585)	Low	Low	Low	Low	Low	Low	Unclear
Katisko et al. (31559531)	Low	Unclear	Low	Unclear	Low	Low	Low
Katisko et al. (34187866)	Low	Low	Low	Low	Low	Low	Low
Katzeff et al. (32792518)	Low	Low	Low	Low	Low	Low	Low
Khoonsari et al. (30614806)	Low	Unclear	Low	Unclear	Low	Low	Low
Körtvélyessy et al. (25777512)	Low	Low	Low	Unclear	Low	Low	Low

Study (PMID)	Risk of bias				Applicability concerns		
	**Patient selection**	**Index test**	**Reference standard**	**Flow and timing**	**Patient selection**	**Index test**	**Reference standard**
Li et al. (34826032)	Low	Low	Low	Unclear	Low	Low	Unclear
Ljubenkov et al. (31620075)	Low	Low	Low	Unclear	Low	Low	Unclear
Ljubenkov et al. (34559230)	Low	Low	Low	Unclear	Low	Low	Low
Lok et al. (37268663)	Low	Unclear	Low	Low	Low	Low	Low
Longobardi et al. (35159272)	Low	Low	Low	Low	Low	Low	Low
Maetzler et al. (21593566)	Low	Low	Low	Low	Low	Low	Low
Magdalinou et al. (25589779)	Low	Low	Low	Low	Low	Low	Low
Meda et al. (37476993)	Low	Low	Low	Low	Low	Low	Unclear
Miller et al. (23543794)	Low	Low	Low	Unclear	Low	Low	Unclear
Minthon et al. (9213068)	Low	Low	Low	Low	Low	Low	Low
Mohaupt et al. (39736793)	Low	Low	Low	Low	Low	Low	Unclear
Oeckl et al. (30224549)	Low	Low	Low	Low	Low	Low	Low
Oeckl et al. (30594925)	Low	Low	Low	Low	Low	Low	Low
Oeckl et al. (35477892)	Low	Low	Low	Low	Low	Low	Low
Olğun et al. (38803182)	Low	Low	Low	Low	Low	Low	Low
Pawlowski et al. (29107846)	Low	Unclear	Unclear	Low	Low	Low	Low
Petzold et al. (12505619)	Low	Low	Low	Low	Low	Low	Low
Phan et al. (32107421)	Low	Low	Low	Low	Low	Low	Low
Phan et al. (35083468)	Low	Low	Low	Low	Low	Low	Unclear
Philips et al. (21107132)	Low	Low	Low	Low	Unclear	Low	Low
Piscopo et al. (23396349)	Unclear	Low	Low	Low	Low	Low	Low
Rentzos et al. (16690997)	Low	Low	Low	Low	Low	Low	Low
Rentzos et al. (16843497)	Low	Low	Low	Low	Low	Low	Low
Roos et al. (30193769)	Low	Low	Low	Low	Low	Low	Low

Study (PMID)	Risk of bias				Applicability concerns		
	Patient selection	Index test	Reference standard	Flow and timing	Patient selection	Index test	Reference standard
Rostgaard et al. (29237796)	Low	Low	Low	Low	Low	Low	Low
Sanchez et al. (38105605)	Low	Low	Low	Low	Low	Low	Low
Saraceno et al. (39125924)	Low	Low	Low	Low	Low	Low	Low
Sarto et al. (36450604)	Low	Low	Low	Low	Low	Low	Low
Schofield et al. (20858962)	Low	Low	Low	Low	Low	Low	Low
Schröder et al. (36358351)	Unclear	Low	Low	Low	Low	Low	Low
Sheinerman et al. (29121998)	Low	Low	Low	Low	Low	Low	Low
Sirisi et al. (35766328)	High	Low	Low	Low	High	Low	Low
Sjögren et al. (11589911)	Low	Low	Low	Low	Low	Low	Low
Sjögren et al. (15258209)	Low	Low	Low	Low	Low	Low	Low
Sleegers et al. (19288468)	Unclear	Low	Unclear	Low	Low	Low	Unclear
Sogorb-Esteve et al. (34542074)	Low	Low	Low	Unclear	Unclear	Low	Low
Sogorb-Esteve et al. (34602080)	Low	Low	Low	Unclear	Low	Low	Low
Steinacker et al. (29142138)	High	Low	Unclear	Low	High	Low	Low
Sudre et al. (31835286)	Low	Low	Low	Low	Low	Low	Low
Taipa et al. (30711675)	Low	Low	Low	Low	Low	Low	Unclear
Teunissen et al. (27239539)	Low	Low	Low	Low	Low	Low	Unclear
Thijssen et al. (35603139)	Low	Low	Low	Low	Low	Low	Low
Toft et al. (36815874)	Low	Low	Low	Low	Low	Low	Unclear
Ullgren et al. (37968725)	Low	Low	Low	Low	Low	Low	Low
Van der Ende et al. (31019994)	Low	Low	Low	Low	Low	Low	Unclear
Van der Ende et al. (33896652)	Low	Low	Low	Low	Low	Low	Low

**Study** (PMID)	**Risk of bias**				**Applicability concerns**		
	**Patient selection**	**Index test**	**Reference standard**	**Flow and timing**	**Patient selection**	**Index test**	**Reference standard**
Van der Ende et al. (34633446)	Low	Low	Low	Low	Low	Low	Unclear
Van der Ende et al. (36064709)	Low	Low	Low	Low	Low	Low	Low
Vieira et al. (33711696)	Low	Low	Low	Low	Low	Low	Low
Villar-Piqué et al. (31299989)	Low	Low	Low	Unclear	Low	Low	Low
Wang et al. (20028451)	Low	Unclear	Low	Unclear	Low	Low	Low
Wang et al. (36575499)	Low	Low	Low	Low	Low	Low	Low
Woollacott et al. (36245297)	Low	Low	Low	Low	Low	Low	Low
Zhou et al. (36989373)	Low	Low	Low	Low	Low	Low	Low
Zhu et al. (34893073)	Low	Low	Low	Low	Low	Low	Low

Note: Studies highlighted in red were excluded from the systematic review due to a lack of defined diagnostic criteria or clear inclusion/exclusion criteria for FTD patients.
