# Supplementary material for: Immunological Fluid Biomarkers in Frontotemporal Dementia: A Systematic Review
Source: Biomolecules. 2025 Mar 24;15(4):473. doi: 10.3390/biom15040473 (PMC12025258; doi:10.3390/biom15040473)
Supplement: Supplementary file 1 [file biomolecules-15-00473-s001.zip › Supplementary Table S2 - Biomarkers in Clinical Groups.pdf]

Supplementary Table S2. Comparative analysis of immune markers in clinical groups

Reported change in CSF levels	**FTD versus AD patients**<br>CSF-based immune markers
↑↑	No data
↑	GFAP[18],[29],[27],[28] GFAP ratio CSF/Serum[27] AB42/YKL-40 ratio[34] SERPING1[56] SPARC[56]
↓↓	No data
↓	PGRN[156],[157] SPP1[158] SST[46] OPTN[158] Cystatin C[56],[157]
→→	YKL-40[29],[31],[32],[33],[34],[36],[53],[54],[56] MCP1/CCL2[50],[52],[53]
→	CHIT1[29] TREM2[66] CXCL10[50] CXCL12[71] IFN-y[61] IL-12[61],[159] Leukocytes[69] G-CSF[61] GM-CSF[61] HGF[52] IL-10[61] IL-13[61] IL-17[61] IL-2[61] IL-5[61] IL-6[61] IL-7[61] TGF-β[52] S100B[119] SST[45] Complement Factor D[8],[115] DKK-3[73] CCL11[61] IL-1 Ra[61] IL-11[117] IL-4[61] IL-8[50] IL-9[61] KYNA[75] SORL1[120] CCR7- CD45RA+ CD4+[67] CCR7- CD45RA+ CD8+[67] CCR7- CD45RO+ CD8+[67] CCR7+ CD45RA+ CD4+[67] CCR7+ CD45RA+ CD8+[67] CCR7+ CD45RO+ CD4+[67] CCR7+ CD45RO+ CD8+[67] CD45RA- CD45RO- CD4+[67] CD45RA- CD45RO- CD8+[67] CD45RA- CD45RO+ CD4+[67] CD45RA- CD45RO+ CD8+[67] CD45RA+ CD45RO- CD4+[67] CD45RA+ CD45RO- CD8+[67] CD45RA+ CD45RO+ CD4+[67] CD45RA+ CD45RO+ CD8+[67] CD56+ CD3+[67] CD56+ CSF+[67] CD69+ CD3+[67] CD8+[67] CD4+[67] CD3+ CD14-[67] CD14+ CD3-[67] CD14+ CD3+[67] CD19+[67] HLA-DR CD14+[67] HLA-DR CD3+[67] NPY[45]
Contradictory	IL-15[61,101]
Reported change in blood levels	Blood-based immune markers
↑↑	No data
↑	No data
↓↓	GFAP[16],[18],[19],[20],[24],[25],[27],[100],[80]
↓	Orexin[160] PGRN[39] OPTN[158] GFAP ratio Serum/CSF[27] BACE-1[24]
→→	CXCL10[50],[51],
→	PGRN[39],[41] FGF-2[61] TNFα[86] YKL-40[16],[91] miRNA Anti-TDP-43[96] APN[88] CD14[89] CRP[89] IL-1[86] Basophils[93] NOD2[99] SPP1[99] TREM2[86] Autoimmune disease immunopositivity[161] IL-8[50] MCP1/CCL2[50]
Contradictory	No data
Reported change in CSF levels	**FTD versus ALS patients**<br>CSF-based immune markers
↑↑	No data
↑	IL-15[102]
↓↓	No data
↓	CXCL12[71] NFL/YKL-40[35] sAPPb/YKL-40[35] IgG[102] CHIT1[21]
→→	No data

→	KYNA[75] OCB[102] WBC[102] C4[102]
Contradictory	No data
Reported change in blood levels	Blood-based immune markers
↑↑	No data
↑	HERV-K[90]
↓↓	No data
↓	Anti-TDP-43[96] C4[102] NOD2[99]
→→	No data
→	APN[88] C3[102] Leucocytes[102] GFAP[21] PROS-1[78] GSN[78] SPP1[99] Neutrophils[102] hsCRP[102] IgG[102] IgA[102] IgM[102]
Contradictory	No data

FTD subtype comparisons	**FTD phenotypes** CSF-based immune markers		
	**Increased**	**Decreased**	**No change**
FTD (NOS) vs bvFTD			CHIT1[29] GFAP[22] YKL-40[29]
FTD (NOS) vs nfvPPA			CHIT1[29] YKL-40[29]
FTD (not specified) vs svPPA			CHIT1[29] YKL-40[29]
FTD (NOS) vs lpPPA			CHIT1[29] YKL-40[29]
FTD (NOS) vs PPA (NOS)			CHIT1[29] GFAP[22] YKL-40[29]
bvFTD vs nfvPPA	Gal-3[32]		CHIT1[29] GFAP[18] YKL-40[29],[36]
bvFTD vs svPPA	Gal-3[32]		YKL-40[29],[36] CHIT1[29] Monocytes[162] CD14+CD16- monocytes[162] CD14+CD16+ monocytes[162] CXCL10[50] MCP1/CCL2[50] IL-8[50]
bvFTD vs lpPPA			YKL-40[29] CHIT1[29] GFAP[18] Monocytes[162] CD14+CD16- monocytes[162] CD14+CD16+ monocytes[162]
bvFTD vs PPA (NOS)			YKL-40[29] CHIT1[29] CXCL10[50] MCP1/CCL2[50] IL-8[50] GFAP[22],[29] CHIT1[29]
nfvPPA vs. svPPA			YKL-40[29],[36] CHIT1[29] Gal-3[32] CCL2[43] CCL3[43] CCL4[43] CCL8[43] CCL11[43] CCL19[43] CCL23[43] CCL25[43] CCL28[43] CXCL5[43] CXCL6[43] CXCL9[43] CXCL10[43] CXCL11[43] IL-8[43]
nfvPPA vs lpPPA		CX3CL1[43] CXCL1[43] CCL2[43]	YKL-40[29] CHIT1[29] GFAP[18] CCL3[43] CCL4[43] CCL8[43] CCL11[43] CCL23[43] CCL25[43] CCL28[43] CXCL6[43] CXCL9[43] CXCL10[43] CXCL11[43] IL-8[43]

FTD subtype comparisons	Increased	Decreased	No change
		CCL19[43]<br>CXCL5[43]	
nfvPPA vs. PPA (NOS)			YKL-40[29] CHIT1[29]
svPPA vs. lpPPA			YKL-40[29] CHIT1[29] CCL19[43] Monocytes[162] CD14+CD16- monocytes[162] CD14+CD16+ monocytes[162]
svPPA vs. PPA (NOS)			CXCL10[50] MCP1/CCL2[50] IL-8[50]
**FTD subtype comparisons**	**Blood-based immune markers**		
	**Increased**	**Decreased**	**No change**
FTD (NOS) vs bvFTD			PGRN[41]
FTD (NOS) vs svPPA			PGRN[41]
FTD (NOS) vs PPA(NOS)			PGRN[39]
bvFTD vs nfvPPA			GFAP[18] Gal-3[32] PGRN[41] IL-6[41]
bvFTD vs svPPA			Gal-3[32] Monocytes[162] CD14+CD16- monocytes[162] CD14+CD16+ monocytes[162] PGRN[41] IL-6[41] CXCL10[50] MCP1/CCL2[50] IL-8[50]
bvFTD vs lpPPA			GFAP[18] Monocytes[162] CD14+CD16- monocytes[162] CD14+CD16+ monocytes[162]
bvFTD vs PPA(NOS)			CXCL10[50] MCP1/CCL2[50] IL-8[50]
nfvPPA vs svPPA			Gal-3[32] PGRN[41] IL-6[41]
nfvPPA vs lpPPA	CCL3[43]<br>CCL4[43]<br>CCL19[43]		GFAP[18]
svPPA vs lpPPA	CCL3[43]<br>CCL4[43]<br>CCL19[43]	CCL3[43]	Monocytes[162] CD14+CD16- monocytes[162] CD14+CD16+ monocytes[162]
svPPA vs PPA (NOS)			CXCL10[50] MCP1/CCL2[50] IL-8[50]
**FTD subtype comparisons**	**FTD genotypes**<br>**CSF-based immune markers**		
	**Increased**	**Decreased**	**No change**
C9ORF72 vs MAPT		Gal-3[32]	TREM2[37] YKL-40[37] CHIT1[37] C1q[8] C3b[8] GFAP[18] YKL-40[29] CHIT1[29] Leukotriens[62] Prostaglandins[62] Tromboxane[62]

GRN vs. C9ORF72	CD166[66] SSP1[66] YKL-40[29] CHIT1[29]		TREM2[37] YKL-40[37] CHIT1[37] TREM2[65] Leukotriens[62] Prostaglandines[62] Tromboxane[62] GFAP[18] Gal-3[32] C1q[8] C3b[8]
MAPT vs GRN	Gal-3[32]	CHIT1[37]	Leukotriens[62] Prostaglandines[62] Tromboxane[62] GFAP[18] TREM2[37] YKL-40[29],[37] C1q[8] C3b[8]
FTD (NOS) vs C9ORF72			Leukotriens[62] Prostaglandines[62] Tromboxane[62] hsCRP[163]
FTD (NOS) vs MAPT		Tromboxane[62]	Leukotriens[62] Prostaglandines[62]
FTD (NOS) vs GRN			Leukotriens[62] Prostaglandines[62] Tromboxane[62]
**FTD subtype comparisons**	**Blood-based immune markers**		
	**Increased**	**Decreased**	**No change**
C9ORF72 vs MAPT		IL-6[41],[82]	TNFα[82] YKL-40[82] C2[8] C3[8] GFAP[18] Gal-3[32]
C9ORF72 vs GRN	PGRN[39],[41] GFAP[18]		C2[8] C3[8] IL-6[41] TNFα[82] IL-6[82] YKL-40[82] C1q[79] C3[79] C4[79] Gal-3[32]
MAPT vs GRN	IL-6[41],[82]		C2[8] C3[8] TNFα[82] YKL-40[82] GFAP[18] Gal-3[32]
FTD (NOS) vs C9ORF72			MCP1/CCL2[116] RANTES[116] IL-10[116] IL-8[116] IL-1B[116] hsCRP[116] Leukocytes[116] BP180[97] BP230[97] Cathepsin S[98] IL-6[41] PGRN[41]
FTD (NOS) vs MAPT			PGRN[41]
FTD (NOS) vs GRN	PGRN[40],[41]		Cathepsin S[98] IL-6[41]
GRN vs sFTD	C3[79]	PGRN[41],[164]	C1q[79] C4[79]
C9orf72 vs sFTD	C3[79]		C1q[79] C4[79]

The table summarizes immune markers quantified in the CSF and blood of FTD patients, comparing them with healthy controls, as well as AD and ALS patients, and examining differences among FTD phenotypes and genotypes. The markers are categorized based on the direction of change and the strength of supporting evidence. Underlined references indicate studies with statistically significant findings. Two vertical arrows signify that at least two studies have reported significantly increased or decreased levels in FTD, while a single vertical arrow represents one study showing significant changes. Biomarkers with one or more studies reporting unchanged levels are indicated by one or two horizontal arrows, respectively. ‘Contradictory’ indicates that both significantly increased and decreased levels have been reported for the same marker. Blood levels refer to measurements in serum, plasma, or, in a few cases, whole blood. Cluster of differentiation (CD) molecules represent immune cell biomarkers isolated from blood, identified as positive or negative for the respective CD markers. NOS: not otherwise specified.
