# Supplementary material for: Immunological Fluid Biomarkers in Frontotemporal Dementia: A Systematic Review
Source: Biomolecules. 2025 Mar 24;15(4):473. doi: 10.3390/biom15040473 (PMC12025258; doi:10.3390/biom15040473)
Supplement: Supplementary file 1 [file biomolecules-15-00473-s001.zip › Supplementary Table S3 - Unfiltered Enrichment Analyses.pdf]

Supplementary Table S3. Unfiltered enrichment analysis

Pathways	Immune markers	Database	Adj. *p*-value
**Increased in CSF**			
Immune System R-HSA-168256	C1QA;CR1;SERPINA1;TGFB1;CXCL8;TREM2;CHIT1;C4A;LGALS3;CXCL10;CCL3;CCL2;CHI3L1;SERPING1	Reactome	1,37E-07
Innate Immune System R-HSA-168249	CHIT1;C1QA;C4A;LGALS3;CR1;SERPINA1;CHI3L1;SERPING1;TREM2	Reactome	1,64E-05
Peptide Ligand-Binding Receptors R-HSA-375276	CXCL10;CXCL8;GPNMB;CCL3;CCL2	Reactome	4,01E-05
Platelet Degranulation R-HSA-114608	SERPINA1;SPARC;TGFB1;SERPING1	Reactome	1,73E-04
Response To Elevated Platelet Cytosolic Ca2+ R-HSA-76005	SERPINA1;SPARC;TGFB1;SERPING1	Reactome	1,80E-04
Signal Transduction R-HSA-162582	CXCL10;SPARC;TGFB1;CXCL8;GPNMB;CCL3;CCL2;NEFL;NGF;MMP10;GFAP	Reactome	2,65E-04
Class A/1 (Rhodopsin-like Receptors) R-HSA-373076	CXCL10;CXCL8;GPNMB;CCL3;CCL2	Reactome	3,06E-04
Signaling By Interleukins R-HSA-449147	CXCL10;TGFB1;CXCL8;CCL3;CCL2	Reactome	1,28E-03
GPCR Ligand Binding R-HSA-500792	CXCL10;CXCL8;GPNMB;CCL3;CCL2	Reactome	1,28E-03
Neutrophil Degranulation R-HSA-6798695	CHIT1;LGALS3;CR1;SERPINA1;CHI3L1	Reactome	1,32E-03
Signaling By GPCR R-HSA-372790	CXCL10;CXCL8;GPNMB;CCL3;CCL2	Reactome	5,23E-03
Cytokine Signaling In Immune System R-HSA-1280215	CXCL10;TGFB1;CXCL8;CCL3;CCL2	Reactome	5,43E-03
Complement and coagulation cascades	C1QA;C4A;CR1;SERPINA1;C9;SERPING1	KEGG Pathways	2,75E-08
Chagas disease	C1QA;TGFB1;CXCL8;CCL3;CCL2	KEGG Pathways	3,03E-06
Coronavirus disease	C1QA;C4A;CXCL10;CXCL8;C9;CCL2	KEGG Pathways	3,84E-06
Cytokine-cytokine receptor interaction	CXCL10;TGFB1;CXCL8;CCL3;CCL2;NGF	KEGG Pathways	9,46E-06
Neutrophil Chemotaxis (GO:0030593)	LGALS3;CXCL10;CXCL8;CCL3;CCL2	Biol. Process (GO)	4,23E-06
Granulocyte Chemotaxis (GO:0071621)	LGALS3;CXCL10;CXCL8;CCL3;CCL2	Biol. Process (GO)	4,23E-06
Neutrophil Migration (GO:1990266)	LGALS3;CXCL10;CXCL8;CCL3;CCL2	Biol. Process (GO)	4,23E-06
Positive Regulation Of ERK1 And ERK2 Cascade (GO:0070374)	TGFB1;GPNMB;CCL3;CCL2;CHI3L1;TREM2	Biol. Process (GO)	5,36E-06
Inflammatory Response (GO:0006954)	CXCL10;TGFB1;CXCL8;CCL3;CCL2;CHI3L1	Biol. Process (GO)	1,56E-05
Regulation Of ERK1 And ERK2 Cascade (GO:0070372)	TGFB1;GPNMB;CCL3;CCL2;CHI3L1;TREM2	Biol. Process (GO)	1,56E-05
Cellular Response To Lipopolysaccharide (GO:0071222)	CXCL10;TGFB1;CXCL8;CCL3;CCL2	Biol. Process (GO)	1,56E-05
Positive Regulation Of MAPK Cascade (GO:0043410)	TGFB1;GPNMB;CCL3;CCL2;CHI3L1;TREM2	Biol. Process (GO)	4,21E-05
Regulation Of Angiogenesis (GO:0045765)	CXCL10;SPARC;CXCL8;GPNMB;CHI3L1	Biol. Process (GO)	8,03E-05
Positive Regulation Of Macromolecule Metabolic Process (GO:0010604)	TGFB1;CXCL8;CCL3;TREM2;NGF	Biol. Process (GO)	7,76E-04
Positive Regulation Of Protein Phosphorylation (GO:0001934)	TGFB1;GPNMB;CHI3L1;TREM2;NGF	Biol. Process (GO)	8,43E-04
Negative Regulation Of Cell Population Proliferation (GO:0008285)	TGFB1;CXCL8;GPNMB;TREM2;NGF	Biol. Process (GO)	8,43E-04
Regulation Of Cell Population Proliferation (GO:0042127)	CXCL10;CR1;TGFB1;CXCL8;GPNMB;NGF	Biol. Process (GO)	1,80E-03
Positive Regulation Of Gene Expression (GO:0010628)	TGFB1;CXCL8;CCL3;TREM2;NGF	Biol. Process (GO)	1,86E-03
Negative Regulation Of Cellular Process (GO:0048523)	TGFB1;CXCL8;GPNMB;TREM2;NGF	Biol. Process (GO)	2,64E-03
Regulation Of Gene Expression (GO:0010468)	TGFB1;CXCL8;CCL3;TREM2;NGF	Biol. Process (GO)	2,60E-02
Secretory Granule Lumen (GO:0034774)	CHIT1;SERPINA1;SPARC;TGFB1;CHI3L1;SERPING1	Cell. Component (GO)	1,92E-05
Collagen-Containing Extracellular Matrix (GO:0062023)	C1QA;LGALS3;SERPINA1;SPARC;TGFB1;SERPING1	Cell. Component (GO)	2,71E-05
Intracellular Organelle Lumen (GO:0070013)	CHIT1;C4A;SERPINA1;SPARC;TGFB1;SERPING1;NGF	Cell. Component (GO)	2,14E-04
Receptor Ligand Activity (GO:0048018)	LGALS3;CXCL10;TGFB1;GPNMB;CCL3;NGF	Mol. Function (GO)	1,11E-05
Cytokine Activity (GO:0005125)	CXCL10;TGFB1;CXCL8;CCL3;CCL2	Mol. Function (GO)	1,16E-05
**Decreased in CSF**			
Interleukin-10 Signaling R-HSA-6783783	CSF1;IL10RB;CCL5;LIF;IL12B;CXCL1;CCL19	Reactome	2,00E-10
Cytokine Signaling In Immune System R-HSA-1280215	CD40;CSF1;IL10RB;IL15;TNFRSF9;TNFSF12;LIF;FLT3LG;CXCL1;VEGFA;CCL5;IL12B;TNFRSF14;CCL19	Reactome	3,86E-10
Immune System R-HSA-168256	CD274;CD40;GRN;CSF1;IL10RB;IL15;TNFRSF9;TNFSF12;LIF;FLT3LG;CXCL1;VEGFA;PLAU;CD8A;CCL5;IL12B;TNFRSF14;CCL19	Reactome	2,42E-08
Chemokine Receptors Bind Chemokines R-HSA-380108	CXCL6;CXCL11;CCL5;CXCL1;CCL19;CXCL5	Reactome	2,42E-08
Peptide Ligand-Binding Receptors R-HSA-375276	CXCL6;CXCL11;SST;CCL5;CXCL1;CCL19;CXCL5	Reactome	1,45E-06
Signaling By Interleukins R-HSA-449147	CSF1;IL10RB;IL15;CCL5;LIF;IL12B;CXCL1;CCL19;VEGFA	Reactome	1,76E-06
Constitutive Signaling By Aberrant PI3K In Cancer R-HSA-2219530	FGF5;KITLG;FGF19;TGFA;FLT3LG	Reactome	5,83E-06
G Alpha (I) Signaling Events R-HSA-418594	CXCL6;CXCL11;SST;CCL5;CXCL1;CCL19;CXCL5	Reactome	1,72E-05
PI3K/AKT Signaling In Cancer R-HSA-2219528	FGF5;KITLG;FGF19;TGFA;FLT3LG	Reactome	1,72E-05
Signal Transduction R-HSA-162582	CXCL6;CD274;TGFA;FLT3LG;CXCL1;CXCL5;VEGFA;FGF5;CXCL11;KITLG;SST;CCL5;FGF19;DNER;TNFSF10;CCL19	Reactome	1,72E-05
PI5P, PP2A And IER3 Regulate PI3K/AKT Signaling R-HSA-6811558	FGF5;KITLG;FGF19;TGFA;FLT3LG	Reactome	1,72E-05
Class A/1 (Rhodopsin-like Receptors) R-HSA-373076	CXCL6;CXCL11;SST;CCL5;CXCL1;CCL19;CXCL5	Reactome	1,93E-05
Negative Regulation Of PI3K/AKT Network R-HSA-199418	FGF5;KITLG;FGF19;TGFA;FLT3LG	Reactome	2,00E-05
GPCR Ligand Binding R-HSA-500792	CXCL6;CXCL11;SST;CCL5;CXCL1;CCL19;CXCL5	Reactome	1,51E-04
Signaling By Receptor Tyrosine Kinases R-HSA-9006934	FGF5;CD274;KITLG;FGF19;TGFA;FLT3LG;VEGFA	Reactome	2,36E-04
TNFR2 Non-Canonical NF-kB Pathway R-HSA-5668541	CD40;TNFRSF9;TNFSF12;TNFRSF14	Reactome	2,97E-04
GPCR Downstream Signaling R-HSA-388396	CXCL6;CXCL11;SST;CCL5;CXCL1;CCL19;CXCL5	Reactome	7,54E-04
PIP3 Activates AKT Signaling R-HSA-1257604	FGF5;KITLG;FGF19;TGFA;FLT3LG	Reactome	7,54E-04
RAF/MAP Kinase Cascade R-HSA-5673001	FGF5;KITLG;FGF19;TGFA;FLT3LG	Reactome	7,54E-04

MAPK1/MAPK3 Signaling R-HSA-5684996	FGF5;KITLG;FGF19;TGFA;FLT3LG	Reactome	7,60E-04
Signaling By GPCR R-HSA-372790	CXCL6;CXCL11;SST;CCL5;CXCL1;CCL19;CXCL5	Reactome	1,04E-03
Intracellular Signaling By Second Messengers R-HSA-9006925	FGF5;KITLG;FGF19;TGFA;FLT3LG	Reactome	1,12E-03
MAPK Family Signaling Cascades R-HSA-5683057	FGF5;KITLG;FGF19;TGFA;FLT3LG	Reactome	1,30E-03
Diseases Of Signal Transduction By Growth Factor Receptors And Second Messengers R-HSA-5663202	FGF5;KITLG;FGF19;TGFA;FLT3LG	Reactome	4,11E-03
Cytokine-cytokine receptor interaction	CXCL6;CCL24;CD40;CSF1;IL10RB;IL15;TNFRSF9;TNFSF12;LIF;CXCL1;CXCL5;CXCL11;CCL5;TNFSF10;IL12B;TNFRSF14;CCL19	KEGG Pathways	1,65E-20
Viral protein interaction with cytokine and cytokine receptor	CXCL6;CCL24;CXCL11;CSF1;IL10RB;CCL5;TNFSF10;CXCL1;TNFRSF14;CCL19;CXCL5	KEGG Pathways	5,18E-16
Rheumatoid arthritis	CXCL6;CSF1;IL15;CCL5;CXCL1;CXCL5;VEGFA	KEGG Pathways	7,45E-09
TNF signaling pathway	CXCL6;CSF1;IL15;CCL5;LIF;CXCL1;CXCL5	KEGG Pathways	2,09E-08
Ras signaling pathway	FGF5;KITLG;CSF1;HGF;FGF19;TGFA;FLT3LG;VEGFA	KEGG Pathways	1,03E-07
MAPK signaling pathway	FGF5;KITLG;CSF1;HGF;FGF19;TGFA;FLT3LG;VEGFA	KEGG Pathways	5,10E-07
Chemokine signaling pathway	CXCL6;CCL24;CXCL11;CCL5;CXCL1;CCL19;CXCL5	KEGG Pathways	5,10E-07
PI3K-Akt signaling pathway	FGF5;KITLG;CSF1;HGF;FGF19;TGFA;FLT3LG;VEGFA	KEGG Pathways	1,71E-06
Pathways in cancer	FGF5;KITLG;IL15;HGF;FGF19;TGFA;IL12B;FLT3LG;VEGFA	KEGG Pathways	2,57E-06
Hematopoietic cell lineage	KITLG;CSF1;CD5;CD8A;FLT3LG	KEGG Pathways	7,67E-06
Rap1 signaling pathway	FGF5;KITLG;CSF1;HGF;FGF19;VEGFA	KEGG Pathways	1,41E-05
Lipid and atherosclerosis	CD40;CCL5;TNFSF10;IL12B;CXCL1	KEGG Pathways	2,42E-04
Cell adhesion molecules	CD274;CD40;CD6;CD8A	KEGG Pathways	7,80E-04
Positive Regulation Of Cellular Process (GO:0048522)	GRN;CSF1;IL15;LIF;TGFA;FLT3LG;CXCL5;VEGFA;FGF5;KITLG;CCL5;FGF19;IL12B;CCL19	Biol. Process (GO)	3,22E-10
Chemokine-Mediated Signaling Pathway (GO:0070098)	CXCL6;CCL24;CXCL11;CCL5;CXCL1;CCL19;CXCL5	Biol. Process (GO)	1,95E-09
Cellular Response To Chemokine (GO:1990869)	CXCL6;CCL24;CXCL11;CCL5;CXCL1;CCL19;CXCL5	Biol. Process (GO)	1,95E-09
Regulation Of Cell Population Proliferation (GO:0042127)	CSF1;IL15;TNFRSF9;LIF;TGFA;FLT3LG;CXCL1;CXCL5;VEGFA;FGF5;CXCL11;KITLG;SST;FGF19	Biol. Process (GO)	2,39E-09
Positive Regulation Of Cell Population Proliferation (GO:0008284)	FGF5;GRN;KITLG;CSF1;IL15;CCL5;FGF19;LIF;TGFA;FLT3LG;CXCL5;VEGFA	Biol. Process (GO)	2,39E-09
Neutrophil Chemotaxis (GO:0030593)	CXCL6;CCL24;CXCL11;CCL5;CXCL1;CCL19;CXCL5	Biol. Process (GO)	3,39E-09
Granulocyte Chemotaxis (GO:0071621)	CXCL6;CCL24;CXCL11;CCL5;CXCL1;CCL19;CXCL5	Biol. Process (GO)	3,79E-09
Positive Regulation Of Lymphocyte Proliferation (GO:0050671)	CD274;CD40;CD6;IL15;CCL5;IL12B;CCL19	Biol. Process (GO)	3,79E-09
Positive Regulation Of Peptidyl-Tyrosine Phosphorylation (GO:0050731)	CD40;IL15;CCL5;HGF;LIF;IL12B;TNFRSF14;VEGFA	Biol. Process (GO)	4,00E-09
Neutrophil Migration (GO:1990266)	CXCL6;CCL24;CXCL11;CCL5;CXCL1;CCL19;CXCL5	Biol. Process (GO)	4,04E-09
Inflammatory Response (GO:0006954)	CXCL6;CCL24;CD40;CXCL11;IL10RB;CCL5;CXCL1;CCL19;CXCL5	Biol. Process (GO)	1,29E-08
Cytokine-Mediated Signaling Pathway (GO:0019221)	CXCL6;CCL24;CXCL11;IL15;CCL5;IL12B;CXCL1;CCL19;CXCL5	Biol. Process (GO)	2,52E-08
Positive Regulation Of Tyrosine Phosphorylation Of STAT Protein (GO:0042531)	CD40;IL15;CCL5;LIF;IL12B;VEGFA	Biol. Process (GO)	2,65E-08
Positive Regulation Of Protein Phosphorylation (GO:0001934)	FGF5;CD40;CSF1;IL15;HGF;FGF19;LIF;TNFRSF14;CCL19;VEGFA	Biol. Process (GO)	2,99E-08
Regulation Of Tyrosine Phosphorylation Of STAT Protein (GO:0042509)	CD40;IL15;CCL5;LIF;IL12B;VEGFA	Biol. Process (GO)	6,06E-08
Positive Regulation Of T Cell Proliferation (GO:0042102)	CD274;CD6;IL15;CCL5;IL12B;CCL19	Biol. Process (GO)	6,06E-08
Cellular Response To Lipopolysaccharide (GO:0071222)	CXCL6;CD274;CXCL11;CD6;CCL5;CXCL1;CXCL5	Biol. Process (GO)	7,02E-08
Regulation Of Cell Migration (GO:0030334)	FGF5;CCL24;GRN;CSF1;PLAU;SST;CCL5;HGF;FGF19;VEGFA	Biol. Process (GO)	9,06E-08
Positive Regulation Of Cell Motility (GO:2000147)	CCL24;GRN;CSF1;PLAU;CCL5;HGF;CCL19;VEGFA	Biol. Process (GO)	1,30E-07
Regulation Of T Cell Proliferation (GO:0042129)	CD274;CD6;IL15;CCL5;IL12B;CCL19	Biol. Process (GO)	1,37E-07
Regulation Of Peptidyl-Tyrosine Phosphorylation (GO:0050730)	IL15;HGF;LIF;IL12B;TNFRSF14;VEGFA	Biol. Process (GO)	3,37E-07
Positive Regulation Of T Cell Activation (GO:0050870)	CD274;CD6;IL15;CCL5;IL12B;CCL19	Biol. Process (GO)	9,15E-07
Macrophage Differentiation (GO:0030225)	CSF1;IL15;LIF;VEGFA	Biol. Process (GO)	1,38E-06
Positive Regulation Of MAPK Cascade (GO:0043410)	CCL24;CD40;CCL5;FGF19;LIF;TGFA;CCL19;VEGFA	Biol. Process (GO)	1,43E-06
Positive Regulation Of Cytokine Production (GO:0001819)	CD274;CD40;CD6;IL15;IL12B;TNFRSF14;CCL19;CD244	Biol. Process (GO)	1,76E-06
Myeloid Leukocyte Differentiation (GO:0002573)	CSF1;IL15;LIF;CCL19;VEGFA	Biol. Process (GO)	3,88E-06
Positive Regulation Of Response To External Stimulus (GO:0032103)	GRN;PLAU;IL15;CCL5;IL12B;CCL19	Biol. Process (GO)	6,78E-06
Response To Lipopolysaccharide (GO:0032496)	CXCL6;CD274;CXCL11;CD6;CXCL1;CXCL5	Biol. Process (GO)	7,60E-06
Positive Regulation Of Cell Migration (GO:0030335)	CCL24;GRN;CSF1;PLAU;CCL5;HGF;VEGFA	Biol. Process (GO)	9,22E-06
Cellular Response To Molecule Of Bacterial Origin (GO:0071219)	CXCL6;CD274;CXCL11;CXCL1;CXCL5	Biol. Process (GO)	3,83E-05
Positive Regulation Of Epithelial Cell Proliferation (GO:0050679)	CCL24;GRN;TNFSF12;TGFA;VEGFA	Biol. Process (GO)	4,75E-05
Positive Regulation Of Intracellular Signal Transduction (GO:1902533)	CD40;CCL5;HGF;TNFSF10;LIF;IL12B;CCL19;VEGFA	Biol. Process (GO)	5,39E-05
Positive Regulation Of Phosphorylation (GO:0042327)	FGF5;CD40;CCL5;HGF;FGF19;VEGFA	Biol. Process (GO)	5,39E-05
Transmembrane Receptor Protein Tyrosine Kinase Signaling Pathway (GO:0007169)	FGF5;CSF1;CD8A;HGF;FGF19;VEGFA	Biol. Process (GO)	1,58E-04
Positive Regulation Of Protein Modification Process (GO:0031401)	FGF5;CD40;HGF;FGF19;VEGFA	Biol. Process (GO)	5,87E-04
Cellular Response To Lipid (GO:0071396)	CXCL6;CD274;CXCL11;CXCL1;CXCL5	Biol. Process (GO)	6,01E-04
Regulation Of Protein Phosphorylation (GO:0001932)	FGF5;CD40;HGF;FGF19;VEGFA	Biol. Process (GO)	1,01E-03
Cellular Response To Cytokine Stimulus (GO:0071345)	CCL24;CD40;CCL5;IL12B;CCL19	Biol. Process (GO)	1,87E-03
Cellular Response To Oxygen-Containing Compound (GO:1901701)	CXCL6;CD274;CXCL11;CXCL1;CXCL5	Biol. Process (GO)	4,52E-03
Receptor Ligand Activity (GO:0048018)	CCL24;GRN;CSF1;IL15;HGF;TNFSF12;LIF;TGFA;CXCL1;VEGFA;FGF5;KITLG;SST;CCL5;FGF19;IL12B	Mol. Function (GO)	1,60E-18
Cytokine Activity (GO:0005125)	CXCL6;CCL24;CSF1;IL15;LIF;CXCL1;CXCL5;VEGFA;CXCL11;KITLG;CCL5;IL12B;CCL19	Mol. Function (GO)	3,77E-17
Growth Factor Activity (GO:0008083)	FGF5;GRN;CSF1;HGF;FGF19;LIF;TGFA;IL12B;CXCL1;VEGFA	Mol. Function (GO)	2,68E-15
Chemokine Activity (GO:0008009)	CXCL6;CCL24;CXCL11;CCL5;CXCL1;CCL19;CXCL5	Mol. Function (GO)	1,45E-11
Chemokine Receptor Binding (GO:0042379)	CXCL6;CCL24;CXCL11;CCL5;CXCL1;CCL19;CXCL5	Mol. Function (GO)	2,16E-11
Cytokine Receptor Binding (GO:0005126)	KITLG;CSF1;CCL5;LIF;IL12B;CCL19;VEGFA	Mol. Function (GO)	2,71E-09
Growth Factor Receptor Binding (GO:0070851)	FGF5;FGF19;TGFA;IL12B;VEGFA	Mol. Function (GO)	4,08E-06

Increased in blood			
Interleukin-10 Signaling R-HSA-6783783	IL10;CXCL10;IL6;IL1B;CCL4;CCL3;CCL2;IL12B;TNFRSF1B;TNF;TNFRSF1A	Reactome	2,28E-19
Cytokine Signaling In Immune System R-HSA-1280215	IL10;CCL11;IL15;TNFSF12;IL16;TNFRSF1B;TNF;IL2;TNFSF13B;TNFRSF1A;CXCL10;IL6;IL7;IL2RA;IL1B;CCL4;CCL3;CCL2;IL12B;S100A12;TNFRSF8	Reactome	2,28E-19
Signaling By Interleukins R-HSA-449147	IL10;CCL11;IL15;IL16;TNFRSF1B;TNF;IL2;TNFRSF1A;CXCL10;IL6;IL7;IL2RA;IL1B;CCL4;CCL3;CCL2;IL12B;S100A12	Reactome	1,71E-18
Immune System R-HSA-168256	CCL11;TNF;TNFSF13B;C3;ANPEP;CCL4;CCL3;CCL2;IL12B;S100A12;TNFRSF8;IL10;IL15;TNFSF12;IL16;TNFRSF1B;IL2;TNFRSF1A;CXCL10;IL6;MYH2;SLPI;IL7;IL2RA;IL1B;CHI3L1;S100A8	Reactome	5,31E-18
Interleukin-4 And Interleukin-13 Signaling R-HSA-6785807	IL10;IL6;CCL11;IL1B;CCL2;IL12B;TNFRSF1B;TNF	Reactome	5,81E-10
Chemokine Receptors Bind Chemokines R-HSA-380108	CCL13;CXCL10;CCL11;CCL4;CCL3;CCL2	Reactome	2,07E-08
TNFR2 Non-Canonical NF-kB Pathway R-HSA-5668541	TNFSF12;TNFRSF8;TNFRSF1B;TNF;TNFSF13B;TNFRSF1A	Reactome	6,14E-07
Peptide Ligand-Binding Receptors R-HSA-375276	C3;CCL13;CXCL10;CCL11;CCL4;CCL3;CCL2	Reactome	1,26E-06
TNFs Bind Their Physiological Receptors R-HSA-5669034	TNFRSF8;TNFRSF1B;TNFSF13B;TNFRSF1A	Reactome	3,73E-06
Class A/1 (Rhodopsin-like Receptors) R-HSA-373076	C3;CCL13;CXCL10;CCL11;CCL4;CCL3;CCL2	Reactome	2,87E-05
Leishmania Infection R-HSA-9658195	C3;IL10;CD163;IL6;MYH2;IL1B	Reactome	7,28E-05
GPCR Ligand Binding R-HSA-500792	C3;CCL13;CXCL10;CCL11;CCL4;CCL3;CCL2	Reactome	2,19E-04
Neutrophil Degranulation R-HSA-6798695	C3;SLPI;ANPEP;S100A12;CHI3L1;TNFRSF1B;S100A8	Reactome	2,33E-04
Signal Transduction R-HSA-162582	CCL13;CCL11;TNF;IL2;GFAP;TNFRSF1A;C3;CXCL10;IL6;IL2RA;CCL4;CCL3;CCL2;S100A8	Reactome	7,49E-04
Innate Immune System R-HSA-168249	C3;MYH2;SLPI;ANPEP;IL1B;S100A12;CHI3L1;TNFRSF1B;S100A8	Reactome	7,88E-04
Signaling By GPCR R-HSA-372790	C3;CCL13;CXCL10;CCL11;CCL4;CCL3;CCL2	Reactome	1,59E-03
Infectious Disease R-HSA-5663205	C3;IL10;CD163;IL6;MYH2;IL1B	Reactome	3,11E-02
Disease R-HSA-1643685	C3;IL10;CD163;IL6;MYH2;SLPI;IL1B;S100A8	Reactome	4,51E-02
Cytokine-cytokine receptor interaction	CCL13;CCL11;TNF;TNFSF13B;CCL8;CCL4;CCL3;CCL2;IL12B;TNFRSF8;CCL18;IL10;IL15;TNFSF12;IL16;TNFRSF1B;IL2;TNFRSF1A;CXCL10;IL6;IL7;IL2RA;IL1B;IL17A;CCL26	KEGG Pathways	2,45E-35
Viral protein interaction with cytokine and cytokine receptor	IL10;CCL13;CCL11;TNFRSF1B;TNF;IL2;TNFRSF1A;CXCL10;IL6;CCL8;IL2RA;CCL4;CCL3;CCL2;CCL18;CCL26	KEGG Pathways	4,00E-26
Chagas disease	C3;IL10;IL6;IL1B;CCL3;CCL2;IL12B;TNF;IL2;TNFRSF1A	KEGG Pathways	9,64E-14
Rheumatoid arthritis	IL6;IL15;IL1B;CCL3;CCL2;TNF;TNFSF13B;IL17A	KEGG Pathways	1,39E-10
IL-17 signaling pathway	CXCL10;IL6;CCL11;IL1B;CCL2;TNF;S100A8;IL17A	KEGG Pathways	1,39E-10
Coronavirus disease	C3;CXCL10;IL6;IL1B;CCL2;IL12B;TNF;IL2;C2;TNFRSF1A	KEGG Pathways	1,96E-10
TNF signaling pathway	CXCL10;IL6;IL15;IL1B;CCL2;TNFRSF1B;TNF;TNFRSF1A	KEGG Pathways	3,64E-10
Inflammatory bowel disease	IL10;IL6;IL1B;IL12B;TNF;IL2;IL17A	KEGG Pathways	3,64E-10
African trypanosomiasis	IL10;IL6;IL1B;IL12B;APOL1;TNF	KEGG Pathways	6,69E-10
Chemokine signaling pathway	CCL13;CXCL10;CCL11;CCL8;CCL4;CCL3;CCL2;CCL18;CCL26	KEGG Pathways	6,69E-10
Pertussis	C3;IL10;IL6;IL1B;IL12B;TNF;C2	KEGG Pathways	8,19E-10
JAK-STAT signaling pathway	IL10;IL6;IL15;IL7;IL2RA;IL12B;IL2;GFAP	KEGG Pathways	4,70E-09
Toll-like receptor signaling pathway	CXCL10;IL6;IL1B;CCL4;CCL3;IL12B;TNF	KEGG Pathways	6,48E-09
Intestinal immune network for IgA production	IL10;IL6;IL15;IL2;TNFSF13B	KEGG Pathways	1,76E-07
Hematopoietic cell lineage	IL6;IL7;ANPEP;IL2RA;IL1B;TNF	KEGG Pathways	1,76E-07
Influenza A	CXCL10;IL6;IL1B;CCL2;IL12B;TNF;TNFRSF1A	KEGG Pathways	1,76E-07
Malaria	IL10;IL6;IL1B;CCL2;TNF	KEGG Pathways	1,93E-07
C-type lectin receptor signaling pathway	IL10;IL6;IL1B;IL12B;TNF;IL2	KEGG Pathways	1,93E-07
NF-kappa B signaling pathway	CCL13;IL1B;CCL4;TNF;TNFSF13B;TNFRSF1A	KEGG Pathways	1,93E-07
Tuberculosis	C3;IL10;IL6;IL1B;IL12B;TNF;TNFRSF1A	KEGG Pathways	1,93E-07
Legionellosis	C3;IL6;IL1B;IL12B;TNF	KEGG Pathways	3,18E-07
Lipid and atherosclerosis	IL6;IL1B;CCL3;CCL2;IL12B;TNF;TNFRSF1A	KEGG Pathways	5,92E-07
Human cytomegalovirus infection	IL6;IL1B;CCL4;CCL3;CCL2;TNF;TNFRSF1A	KEGG Pathways	7,71E-07
Yersinia infection	IL10;IL6;IL1B;CCL2;TNF;IL2	KEGG Pathways	8,09E-07
Leishmaniasis	C3;IL10;IL1B;IL12B;TNF	KEGG Pathways	1,23E-06
Type I diabetes mellitus	IL1B;IL12B;TNF;IL2	KEGG Pathways	4,21E-06
Amoebiasis	IL10;IL6;IL1B;IL12B;TNF	KEGG Pathways	4,31E-06
Th17 cell differentiation	IL6;IL2RA;IL1B;IL2;IL17A	KEGG Pathways	5,28E-06
Human T-cell leukemia virus 1 infection	IL6;IL15;IL2RA;TNF;IL2;TNFRSF1A	KEGG Pathways	9,73E-06
Measles	IL6;IL2RA;IL1B;IL12B;IL2	KEGG Pathways	1,74E-05
Herpes simplex virus 1 infection	C3;IL6;IL1B;CCL2;IL12B;TNF;TNFRSF1A	KEGG Pathways	9,56E-05
Pathways in cancer	IL6;IL15;IL7;IL2RA;IL12B;IL2	KEGG Pathways	9,44E-04
Pathways of neurodegeneration	IL6;IL1B;TNFRSF1B;TNF;TNFRSF1A	KEGG Pathways	3,34E-03
Cytokine-Mediated Signaling Pathway (GO:0019221)	CCL13;CCL11;IL15;TNFRSF1B;TNF;IL2;TNFRSF1A;CXCL10;IL6;CCL8;IL7;IL2RA;IL1B;CCL4;CCL3;CCL2;IL12B;CCL18;IL17A;CCL26	Biol. Process (GO)	1,55E-25
Inflammatory Response (GO:0006954)	CCL13;CCL11;TNF;TNFRSF1A;CXCL10;IL6;CCL8;IL2RA;IL1B;CCL4;CCL3;CCL2;CHI3L1;CCL18;S100A8;CCL26	Biol. Process (GO)	5,96E-19
Monocyte Chemotaxis (GO:0002548)	CCL13;IL6;CCL11;CCL8;CCL4;CCL3;CCL2;S100A12;CCL18;CCL26	Biol. Process (GO)	6,31E-17
Neutrophil Chemotaxis (GO:0030593)	CCL13;CXCL10;CCL11;CCL8;CCL4;CCL3;CCL2;S100A12;CCL18;S100A8;CCL26	Biol. Process (GO)	9,44E-17
Granulocyte Chemotaxis (GO:0071621)	CCL13;CXCL10;CCL11;CCL8;CCL4;CCL3;CCL2;S100A12;CCL18;S100A8;CCL26	Biol. Process (GO)	1,24E-16

Neutrophil Migration (GO:1990266)	CCL13;CXCL10;CCL11;CCL8;CCL4;CCL3;CCL2;S100A12;CCL18;S100A8;CCL26	Biol. Process (GO)	1,93E-16
Cellular Response To Tumor Necrosis Factor (GO:0071356)	CCL13;CCL11;CCL8;CCL4;CCL3;CCL2;CHI3L1;CCL18;TNFRSF1B;TNF;TNFRSF1A;CCL26	Biol. Process (GO)	3,02E-16
Positive Regulation Of Inflammatory Response (GO:0050729)	IL6;IL15;IL1B;CCL3;IL12B;S100A12;IL16;TNF;IL2;S100A8;TNFRSF1A	Biol. Process (GO)	4,43E-15
Eosinophil Chemotaxis (GO:0048245)	CCL13;CCL11;CCL8;CCL4;CCL3;CCL2;CCL18;CCL26	Biol. Process (GO)	4,43E-15
Eosinophil Migration (GO:0072677)	CCL13;CCL11;CCL8;CCL4;CCL3;CCL2;CCL18;CCL26	Biol. Process (GO)	4,43E-15
Lymphocyte Chemotaxis (GO:0048247)	CCL13;CXCL10;CCL11;CCL8;CCL4;CCL3;CCL2;CCL18;CCL26	Biol. Process (GO)	5,69E-15
Cellular Response To Cytokine Stimulus (GO:0071345)	CCL13;CCL11;TNFRSF1A;IL6;CCL8;IL7;IL1B;CCL4;CCL3;CCL2;IL12B;CHI3L1;CCL18;CCL26	Biol. Process (GO)	1,45E-14
Regulation Of Inflammatory Response (GO:0050727)	IL10;IL15;IL16;TNFRSF1B;TNF;IL2;TNFRSF1A;IL6;IL1B;CCL3;IL12B;S100A12;S100A8	Biol. Process (GO)	1,97E-14
Response To Interleukin-1 (GO:0070555)	CCL13;CCL11;CCL8;IL1B;CCL4;CCL3;CCL2;CHI3L1;CCL18;CCL26	Biol. Process (GO)	2,72E-14
Chemokine-Mediated Signaling Pathway (GO:0070098)	CCL13;CXCL10;CCL11;CCL8;CCL4;CCL3;CCL2;CCL18;CCL26	Biol. Process (GO)	5,21E-14
Cellular Response To Chemokine (GO:1990869)	CCL13;CXCL10;CCL11;CCL8;CCL4;CCL3;CCL2;CCL18;CCL26	Biol. Process (GO)	6,80E-14
Positive Regulation Of Response To External Stimulus (GO:0032103)	IL15;IL1B;CCL3;IL12B;S100A12;IL16;TNF;IL2;S100A8;TNFRSF1A;CCL26	Biol. Process (GO)	2,00E-13
Lymphocyte Migration (GO:0072676)	CCL13;CCL11;CCL8;CCL4;CCL3;CCL2;CCL18;CCL26	Biol. Process (GO)	3,13E-13
Positive Regulation Of MAPK Cascade (GO:0043410)	CCL13;CCL11;TNF;IL6;CCL8;IL1B;CCL4;CCL3;CCL2;S100A12;CHI3L1;CCL18;CCL26	Biol. Process (GO)	3,73E-13
Positive Regulation Of Defense Response (GO:0031349)	IL15;IL1B;CCL3;IL12B;S100A12;IL16;TNF;IL2;S100A8;TNFRSF1A	Biol. Process (GO)	9,46E-13
Cellular Response To Interleukin-1 (GO:0071347)	CCL13;CCL11;CCL8;IL1B;CCL4;CCL3;CCL2;CCL18;CCL26	Biol. Process (GO)	1,05E-12
Response To Tumor Necrosis Factor (GO:0034612)	CCL13;CCL11;CCL8;CCL4;CCL3;CCL2;CHI3L1;CCL18;CCL26	Biol. Process (GO)	1,42E-11
Cellular Response To Type II Interferon (GO:0071346)	CCL13;CCL11;CCL8;CCL4;CCL3;CCL2;CCL18;CCL26	Biol. Process (GO)	1,42E-11
Positive Regulation Of Cytokine Production (GO:0001819)	C3;IL10;IL6;IL15;IL7;IL1B;IL12B;CHI3L1;IL16;TNF;IL2;IL17A	Biol. Process (GO)	1,42E-11
Regulation Of ERK1 And ERK2 Cascade (GO:0070372)	CCL13;CCL11;CCL8;IL1B;CCL4;CCL3;CCL2;CHI3L1;CCL18;TNF;CCL26	Biol. Process (GO)	2,05E-11
Positive Regulation Of ERK1 And ERK2 Cascade (GO:0070374)	CCL13;CCL11;CCL8;CCL4;CCL3;CCL2;CHI3L1;CCL18;TNF;CCL26	Biol. Process (GO)	2,99E-11
Response To Type II Interferon (GO:0034341)	CCL13;CCL11;CCL8;CCL4;CCL3;CCL2;CCL18;CCL26	Biol. Process (GO)	6,01E-11
Cellular Response To Lipopolysaccharide (GO:0071222)	IL10;CXCL10;IL6;IL1B;CCL3;CCL2;TNFRSF1B;TNF	Biol. Process (GO)	2,07E-09
Positive Regulation Of Lymphocyte Proliferation (GO:0050671)	IL6;IL15;IL7;IL1B;IL12B;IL2;TNFSF13B	Biol. Process (GO)	2,25E-09
Response To Lipopolysaccharide (GO:0032496)	IL10;CXCL10;IL6;SLPI;IL1B;CCL2;TNFRSF1B;S100A8	Biol. Process (GO)	1,42E-08
Positive Regulation Of Tyrosine Phosphorylation Of STAT Protein (GO:0042531)	IL6;IL15;IL12B;TNF;IL2;TNFRSF1A	Biol. Process (GO)	2,17E-08
Positive Regulation Of Hydrolase Activity (GO:0051345)	CCL13;CCL11;CCL8;CCL4;CCL3;CCL2;CCL18;CCL26	Biol. Process (GO)	2,99E-08
Positive Regulation Of Phagocytosis (GO:0050766)	C3;IL15;IL1B;CCL2;TNF;C2	Biol. Process (GO)	3,50E-08
Regulation Of Tyrosine Phosphorylation Of STAT Protein (GO:0042509)	IL6;IL15;IL12B;TNF;IL2;TNFRSF1A	Biol. Process (GO)	5,56E-08
Regulation Of GTPase Activity (GO:0043087)	CCL13;CCL11;CCL8;CCL4;CCL3;CCL2;CCL18;CCL26	Biol. Process (GO)	1,28E-07
Regulation Of T Cell Proliferation (GO:0042129)	IL10;IL6;IL15;IL1B;IL12B;TNFRSF1B	Biol. Process (GO)	1,48E-07
Positive Regulation Of GTPase Activity (GO:0043547)	CCL13;CCL11;CCL8;CCL4;CCL3;CCL2;CCL18;CCL26	Biol. Process (GO)	2,43E-07
Positive Regulation Of Intracellular Signal Transduction (GO:1902533)	IL6;IL1B;CCL4;CCL3;IL12B;S100A12;CHI3L1;TNF;S100A8;TNFRSF1A	Biol. Process (GO)	7,13E-07
Positive Regulation Of T Cell Activation (GO:0050870)	IL6;IL15;IL7;IL1B;CCL2;IL12B	Biol. Process (GO)	9,53E-07
Positive Regulation Of Chemokine Production (GO:0032722)	IL6;IL7;IL1B;TNF;IL17A	Biol. Process (GO)	9,62E-07
Lipopolysaccharide-Mediated Signaling Pathway (GO:0031663)	IL1B;CCL3;CCL2;TNF	Biol. Process (GO)	9,75E-07
Regulation Of Membrane Protein Ectodomain Proteolysis (GO:0051043)	IL10;IL1B;TNFRSF1B;TNF	Biol. Process (GO)	1,18E-06
Regulation Of Interleukin-6 Production (GO:0032675)	IL10;IL6;IL1B;IL16;TNF;IL17A	Biol. Process (GO)	1,27E-06
Cellular Response To Molecule Of Bacterial Origin (GO:0071219)	IL10;CXCL10;IL6;IL1B;CCL2;TNFRSF1B	Biol. Process (GO)	1,42E-06
Positive Regulation Of Cellular Process (GO:0048522)	IL6;IL15;IL7;IL1B;CLEC11A;IL12B;TNFRSF1B;TNF;IL2;S100A8	Biol. Process (GO)	1,83E-06
Positive Regulation Of Interleukin-1 Production (GO:0032732)	IL6;CCL3;IL16;TNF;IL17A	Biol. Process (GO)	2,07E-06
Regulation Of Tumor Necrosis Factor Production (GO:0032680)	IL10;IL6;CCL3;IL12B;TNFRSF8;IL17A	Biol. Process (GO)	2,09E-06
Positive Regulation Of T Cell Proliferation (GO:0042102)	IL6;IL15;IL1B;IL12B;IL2	Biol. Process (GO)	2,16E-06
Positive Regulation Of Peptidyl-Tyrosine Phosphorylation (GO:0050731)	IL6;IL15;IL12B;TNF;IL2;TNFRSF1A	Biol. Process (GO)	2,31E-06
Response To Molecule Of Bacterial Origin (GO:0002237)	IL10;IL6;SLPI;IL1B;S100A8	Biol. Process (GO)	2,76E-06
Positive Regulation Of Interleukin-6 Production (GO:0032755)	IL6;IL1B;IL16;TNF;IL17A	Biol. Process (GO)	4,35E-06
Positive Regulation Of Tumor Necrosis Factor Production (GO:0032760)	IL6;CCL3;IL12B;TNFRSF8;IL17A	Biol. Process (GO)	4,71E-06
Regulation Of Interleukin-8 Production (GO:0032677)	IL10;IL6;IL1B;CHI3L1;TNF	Biol. Process (GO)	5,60E-06
Positive Regulation Of Tumor Necrosis Factor Superfamily Cytokine Production (GO:1903557)	IL6;CCL3;IL12B;TNFRSF8;IL17A	Biol. Process (GO)	5,86E-06
Regulation Of Cytokine Production (GO:0001817)	IL10;IL6;IL15;IL12B;TNFRSF1B;TNF	Biol. Process (GO)	8,42E-06
Positive Regulation Of Protein Catabolic Process (GO:0045732)	TNFSF12;IL1B;TNFRSF1B;TNF;TNFSF13B	Biol. Process (GO)	9,65E-06
Regulation Of Cell Population Proliferation (GO:0042127)	IL10;BTG3;CXCL10;IL6;IL15;IL7;IL1B;CLEC11A;TNFRSF8;IL2	Biol. Process (GO)	1,32E-05
Positive Regulation Of Protein Metabolic Process (GO:0051247)	IL6;TNFSF12;IL1B;IL12B;TNF;TNFSF13B	Biol. Process (GO)	1,91E-05
Defense Response To Bacterium (GO:0042742)	IL6;SLPI;IL1B;IL12B;S100A12;S100A8	Biol. Process (GO)	2,36E-05
Cellular Response To Lipid (GO:0071396)	IL10;CXCL10;IL6;IL1B;CCL2;TNFRSF1B	Biol. Process (GO)	4,07E-05
Positive Regulation Of DNA-binding Transcription Factor Activity (GO:0051091)	IL10;IL6;IL1B;S100A12;TNF;S100A8	Biol. Process (GO)	5,81E-05
Positive Regulation Of Multicellular Organismal Process (GO:0051240)	IL10;IL6;IL15;IL1B;IL12B;TNF;IL2	Biol. Process (GO)	6,43E-05
Extrinsic Apoptotic Signaling Pathway (GO:0097191)	TNFSF12;TNFRSF1B;TNF;TNFRSF1A	Biol. Process (GO)	6,77E-05
Positive Regulation Of NF-kappaB Transcription Factor Activity (GO:0051092)	IL6;IL1B;S100A12;TNF;S100A8	Biol. Process (GO)	8,27E-05
Positive Regulation Of Cell Population Proliferation (GO:0008284)	IL6;IL15;IL7;IL1B;CLEC11A;TNF;IL2	Biol. Process (GO)	2,24E-04
Regulation Of Angiogenesis (GO:0045765)	CXCL10;IL6;TNFSF12;IL1B;CHI3L1	Biol. Process (GO)	2,81E-04

Positive Regulation Of Protein Phosphorylation (GO:0001934)	C3;IL6;IL15;IL1B;CHI3L1;TNF	Biol. Process (GO)	4,45E-04
Negative Regulation Of Cell Population Proliferation (GO:0008285)	IL10;BTG3;IL6;IL1B;IL12B;TNFRSF8	Biol. Process (GO)	4,45E-04
Cellular Response To Oxygen-Containing Compound (GO:1901701)	IL10;CXCL10;IL6;IL1B;CCL2;TNFRSF1B	Biol. Process (GO)	5,96E-04
Positive Regulation Of Gene Expression (GO:0010628)	IL10;IL6;IL15;IL1B;CCL3;TNF	Biol. Process (GO)	1,27E-03
Negative Regulation Of Apoptotic Process (GO:0043066)	IL10;IL6;IL7;CCL2;TNF;IL2	Biol. Process (GO)	1,29E-03
Regulation Of Apoptotic Process (GO:0042981)	IL10;CXCL10;IL6;IL7;TNFRSF8;TNF;IL2	Biol. Process (GO)	1,49E-03
Positive Regulation Of Cell Migration (GO:0030335)	CCL11;IL1B;CCL3;CCL26	Biol. Process (GO)	5,94E-03
Negative Regulation Of Cellular Process (GO:0048523)	IL10;BTG3;IL6;IL1B;TNFRSF8	Biol. Process (GO)	1,05E-02
Positive Regulation Of DNA-templated Transcription (GO:0045893)	IL10;CXCL10;IL6;IL1B;TNF;TNFRSF1A;IL17A	Biol. Process (GO)	2,30E-02
Regulation Of Gene Expression (GO:0010468)	IL10;IL6;IL1B;CCL3;IL12B;TNF	Biol. Process (GO)	3,61E-02
Secretory Granule Lumen (GO:0034774)	C3;SLPI;S100A12;CHI3L1;S100A8	Cell. Component (GO)	1,62E-02
Cytokine Activity (GO:0005125)	IL10;CCL13;CCL11;IL15;IL16;TNF;IL2;CXCL10;IL6;CCL8;IL7;IL1B;CCL4;CCL3;CCL2;IL12B;CCL18;IL17A;CCL26	Mol. Function (GO)	8,20E-28
Receptor Ligand Activity (GO:0048018)	IL10;CCL11;IL15;TNFSF12;CLEC11A;IL16;TNF;IL2;TNFSF13B;CXCL10;IL6;IL7;IL1B;CCL4;CCL3;IL12B;IL17A;CCL26	Mol. Function (GO)	2,29E-21
Chemokine Activity (GO:0008009)	CCL13;CXCL10;CCL11;CCL8;CCL4;CCL3;CCL2;CCL18;CCL26	Mol. Function (GO)	2,67E-15
Chemokine Receptor Binding (GO:0042379)	CCL13;CXCL10;CCL11;CCL8;CCL4;CCL3;CCL2;CCL18;CCL26	Mol. Function (GO)	4,53E-15
CCR Chemokine Receptor Binding (GO:0048020)	CCL13;CCL11;CCL8;CCL4;CCL3;CCL2;CCL18;CCL26	Mol. Function (GO)	1,72E-13
Growth Factor Activity (GO:0008083)	IL10;IL6;IL7;CLEC11A;IL12B;IL2	Mol. Function (GO)	1,34E-07
Growth Factor Receptor Binding (GO:0070851)	IL10;IL6;IL1B;IL12B;IL2	Mol. Function (GO)	9,57E-06
Cytokine Receptor Binding (GO:0005126)	IL10;IL6;IL1B;IL12B;IL2	Mol. Function (GO)	1,03E-05
**Decreased in blood**			
Immune System R-HSA-168256	IL1A;GRN;IL5;GSN;C1S;CCL20;PROS1;CCL3;CCL19	Reactome	1,09E-06
Signaling By Interleukins R-HSA-449147	IL1A;IL5;CCL20;CCL3;CCL19	Reactome	5,05E-05
Cytokine Signaling In Immune System R-HSA-1280215	IL1A;IL5;CCL20;CCL3;CCL19	Reactome	2,56E-04
Cytokine-cytokine receptor interaction	IL1A;IL5;CCL20;CCL3;CCL19	KEGG Pathways	1,33E-05
Cytokine-Mediated Signaling Pathway (GO:0019221)	IL1A;IL5;CCL20;CCL3;CCL19	Biol. Process (GO)	4,46E-05
Cytokine Activity (GO:0005125)	IL1A;IL5;CCL20;CCL3;CCL19	Mol. Function (GO)	7,01E-07

The table presents pathways overrepresented by immune markers in CSF and blood that exhibit significantly altered levels in FTD compared to healthy controls. The analysis was conducted using the Enrichr platform for enrichment analysis[10]. Pathways were included if they involved at least five significantly upregulated or downregulated immune markers and met a significance threshold of 0.05, with *p*-values adjusted for multiple testing using the Benjamini-Hochberg correction.
